# Molecular Evolutionary Analysis of the Alfin-Like Protein Family in *Arabidopsis lyrata*, *Arabidopsis thaliana*, and *Thellungiella halophila*

**DOI:** 10.1371/journal.pone.0066838

**Published:** 2013-07-01

**Authors:** Yu Song, Jie Gao, Fengxi Yang, Chai-Shian Kua, Jingxin Liu, Charles H. Cannon

**Affiliations:** 1 Key Laboratory of Tropical Forest Ecology, Xishuangbanna Tropical Botanical Garden, Chinese Academy of Sciences, Menglun, Yunnan, People’s Republic of China; 2 Graduate School of the Chinese Academy of Sciences, Beijing People’s Republic of China; 3 Key Laboratory of Bio-Resources and Eco-Environment of Ministry of Education, College of Life Sciences, Sichuan University, Chengdu, Sichuan, People’s Republic of China; 4 Institute of Plant Production and Agroecology in the Tropics and Subtropics (380), Agroecology Section, University of Hohenheim Stuttgart, Germany; 5 Department of Biological Sciences, Texas Tech University, Lubbock, Texas, United States of America; Tel Aviv University, Israel

## Abstract

In previous studies, the *Alfin1* gene, a transcription factor, enhanced salt tolerance in alfalfa, primarily through altering gene expression levels in the root. Here, we examined the molecular evolution of the Alfin-like (AL) proteins in two *Arabidopsis* species (*A. lyrata* and *A. thaliana*) and a salt-tolerant close relative *Thellungiella halophila*. These AL-like proteins could be divided into four groups and the two known DUF3594 and PHD-finger domains had co-evolved within each group of genes, irrespective of species, due to gene duplication events in the common ancestor of all three species while gene loss was observed only in *T. halophila*. To detect whether natural selection acted in the evolution of *AL* genes, we calculated synonymous substitution ratios (*dn/ds*) and codon usage statistics, finding positive selection operated on four branches and significant differences in biased codon usage in the AL family between *T. halophila* and *A. lyrata* or *A. thaliana*. Distinctively, only the AL7 branch was under positive selection on the PHD-finger domain and the three members on the branch showed the smallest difference when codon bias was evaluated among the seven clusters. Functional analysis based on transgenic overexpression lines and T-DNA insertion mutants indicated that salt-stress-induced *AtAL7* could play a negative role in salt tolerance of *A. thaliana*, suggesting that adaptive evolution occurred in the members of *AL* gene family.

## Introduction

Transcriptional control of gene expression underlies different metabolic and developmental processes in plants, including responses to environmental stimuli or stressors [1]–[3]. In the regulation process, *trans*-acting factors sustain, enhance, or repress the expression of genes encoded by the genome. This responsive control over gene expression by these small pieces of genetic material creates a powerful and dynamic mechanism for higher terrestrial plants to adapt to their environment, in addition to more fundamental and genetic responses [4]. Indeed, the evolution of regulating gene may be more powerful and effective than direct structural gene evolution [5]–[7].

Transcription factors can be organized into families based on their characteristic domains, and currently, roughly 84 transcription factor families have been discovered [8]. The Alfin-like protein family was discovered to be a transcription factor in alfalfa (*Medicago sativa*) in the form of a 7S storage protein [9], [10]. The members of this family have two sequences of approximately 130 and 50 conserved amino acid residues at their N-termini and C-termini, respectively, named DUF3594 domain and PHD-finger motif. Originally discovered in a homeodomain protein from *A. thaliana* [11], the latter motif mediates binding capability to specific nuclear protein partners [12], [13]. Besides its protein-protein interaction role, PHD-finger motif also binds to the core consensus *cis*-acting element (C/A) CAC in the promoter of the target gene [14]. Although the former DUF3594 domain is functionally uncharacterized, the highly conserved nature of DUF3594 domain and PHD-finger motif in different species indicates that Alfin-like proteins probably have fundamental biological functions in plants.

Recently, the genes of Alfin-like proteins have been found in many other land plants, such as *Arabidopsis*, rice, and *Glycine max* [2], [15]–[18]. None of AL (Alfin-like protein) protein containing DUF3594 domain were reported in animals, fungi, and prokaryotes [12]. Except for *Alfin1* in alfalfa, the role of these factors in the mediation of biological processes in plants remains unclear. After isolating *Alfin1* cDNA from alfalfa salt-tolerant cells [15], gel retardation assays showed that *Alfin1* protein binds to DNA in a sequence-specific manner [14]. Further, the 35S::*Alfin1* transgenic alfalfa has improved salinity tolerance whereas calli expressing *Alfin1* in the antisense orientation were more sensitive to salt, demonstrating that *Alfin1* functions in salt tolerance in alfalfa [15]. Additionally, the overexpressing lines of *Alfin1* displayed enhanced plant root growth under normal and saline conditions while the antisense transgenic plants grew poorly, suggesting that *Alfin1* expression is essential for normal alfalfa development [19]. Promoter specificity and efficiency of utilization tests indicated Alfin1 protein can bind to the *MsPRP2* promoter directly and enhance its gene expression [19]. In addition, since the orthologs of Alfin1 had been shown to bind to the promoters of *H3K4me2* and *H3K4me3*, the proteins containing the PHD domain might be involved in the process of chromatin regulation both in plants and animals [13], [16], [20].

The evolution of transcription factor genes appears frequently to involve gene duplication and diversification [7]. The slight modification of these *trans*-acting factor genes could in turn regulate large suites of structural genes, giving rise to complex traits. Duplication of regulatory genes has been investigated as a major evolutionary event providing raw material for genetic variations and adaptation [3], [21]–[24]. In order to understand the evolutionary events of *AL* transcription factor genes, we first collected and identified twenty AL family members in three species: *A. lyrata* (7), *A. thaliana* (7), and *Thellungiella halophile* (6). *T. halophila*, a close relative of the other two *Arabidopsis* species [25], exhibits higher resistance to cold, drought, and salinity [26]. The comparison of *AL* genes, transcription factors known to be related to salt-tolerance, found in closely-related species with clear differences in their physiological salt tolerance should provide insight into the molecular mechanism of adaptive evolution in response to strong environmental selection. A phylogenetic tree was constructed to evaluate the evolutionary relationships of all observed *AL* genes and evidence for gene duplication and loss events was examined. Further, we computed synonymous substitution ratios (*dn/ds*) and codon usage statistics to detect positive selection and where it might have occurred on the phylogenetic tree and in which of the two known domains. Finally, overexpression lines and T-DNA insertion mutants of *A. thaliana* for *AL7* gene found to be under positive selection on the PHD finger domain were tested for salt tolerance to understand the physiological effects of the evolutionarily significant gene.

## Materials and Methods

### Sequence Database Searches

The DNA and cDNA sequences of *AL* genes in *A. thaliana* and their ortholog sequences in *A. lyrata* and *T. halophila* were collected from Phytozome Database (http://www.phytozome.net/) and Plant Transcription Factor Database (http://plntfdb.bio.uni-potsdam.de/v3.0/). Then, the databases Pfam (http://pfam.sanger.ac.uk/) and NCBI conserved domain (http://www.ncbi.nlm.nih.gov/Structure/cdd/wrpsb.cgi) were used to confirm the presence of typical domains in their protein structure.

### Sequence Alignment and Phylogenetic Analysis

Full-length amino acid sequences were aligned by the Clustal X 1.83 program [27]. The sequence alignment was further adjusted manually using BioEdit software (http://www.mbio.ncsu.edu/bioedit/bioedit.html ). The amino acid substitution model was calculated by the ModelGenerator v0.84 and the optimal model of “JTT+G” was selected [28]. Phylogenetic relationships were reconstructed using a maximum-likelihood (ML) method in PHYML software with JTT amino acid substitution model [29]. One thousand bootstrap replicates were performed in each analysis to obtain the confidence support. The *Alfin1* gene from *Medicago sativa* was used as an out-group.

### Co-evolution Analysis

The hypothesis that the co-evolved relationships might exist among the domains of proteins during the evolutionary process was tested by Goh and colleagues [30]. Here, we used Goh’s method to test the correlation between every domain-domain pair for the AL family. After the alignments of the DUF3594 domain, PHD-finger domain, and inter-domain, pairwise evolutionary distances for the alignments were calculated using MEGA version 5.0 program [31]. Afterward, we calculated the Pearson correlation coefficients (r) of linear and Mantel analysis between the distance matrices of all potential interacting domains using the SPSS version 13.0 software and PAST Version 2.17 (http://folk.uio.no/ohammer/past/) program (Euclidean, Permutation N: 5000).

### Estimates of Amino Acid Substitution Rates

The nonsynonymous to synonymous substitution rate ratio ω (d_N_/d_S_) is commonly used as an indicator of the selective strength in coding sequences [32]: ω>1 indicates positive selection, ω<1 indicates purifying selection, and ω = 1 indicates neutral evolution. To determine whether positive selection had acted at specific sites in the AL proteins, two codon-based likelihood methods were run using the CODEML package of PAML version 4.2 [33]: site models [34] and branch-site models [35]. For the site models method, six models were explored: the one-ratio model (M_0_), the discrete model (M_3_), the nearly neutral model (M_1a_), the positive-selection model (M_2a_), the beta model (M_7_), and the beta and v model (M_8_). To evaluate variation in selective pressure over a phylogeny, the branch-site models were used to estimate ω under different assumptions. The branch models allow the ω values to vary among branches in the phylogeny and are useful for detecting positive selection acting on particular lineages. To verify which of the models best fitted the data, Likelihood Ratio Tests (LRT) were performed by comparing twice the difference in log likelihood values between pairs of the models using a χ^2^ distribution, with the degrees of freedom equal to the differences in the number of parameters between the models [36]. The Bayes Empirical Bayes (BEB) analysis procedure was used for identifying sites under positive selection with significant LRTs. Each branch group was labeled as foreground in turn as well.

### Synonymous Codon Usage Estimation and Identification of Gene Conversion Events

Codon usage bias, where certain codons are used preferentially, was calculated by the effective number of codons (ENC) [37]. The frequency of G+C at the third synonymous variable codon position (GC3) and codon adaptation index (CAI) were also used to estimate the codon bias. All three analyses were performed on the online platform of EMBOSS explorer (http://emboss.bioinformatics.nl/cgi-bin/emboss). A graphical comparison of ENC and GC3 was used to control for the possible codon bias because it is often associated with GC3 [37]. Additionally, the program GeneConv was used to analyze the possible gene conversion events (http://www.math.wustl.edu/~sawyer/geneconv/).

### Plant Materials and Treatments

*A. thaliana* accessions Columbia (Col)-0 (from *Arabidopsis* Biological Resource Centre at Ohio State University) seeds were germinated. The aboveground parts of three-week-old *A. thaliana* grown at 22°C were harvested and soaked in petri dishes with 25% (w/v) PEG8000 for 0, 1, 2, 4, 12 h; 0 mM, 100 mM, 200 mM, 300 mM NaCl solutions for 4 h; and 300 mM NaCl for 0, 1, 2, 4, 12 h as described in previous studies [38], [39]. All of these plant materials were frozen rapidly in liquid nitrogen and stored at –80°C. For the tolerance assay, three-day-old vector, wild type, mutants and transgenic *Arabidopsis* seedlings were transferred to Murashige & Skoog media (MS) plates supplemented with 150 mM or 200 mM NaCl for 5 days at 22±2°C with 16 h of light and 8 h of darkness [40]. Root length data were analyzed using SPSS version 13.0 software. We first calculated a tolerance index of primary root, resulting in a set of normally distributed data. The index was calculated as: (primary root length at stress condition – primary root length at normal condition)/primary root length at normal condition. We then used one way ANOVA to examine the type of seedlings and salt treatment on the tolerance index of primary root, using Bonferroni multiple comparison tests.

To acquire transgenic plants, the *AtAL7* cDNAs were cloned into the pOCA30 vector, which contained the modified CaMV 35S promoter (*35S-AtAL7*). The fidelity of the construct was confirmed by restriction digestion and sequence analysis. *A. thaliana* plants were transformed by the floral-dip method using *Agrobacterium tumefaciens* strain GV3101 [41]. Transgenic seedlings were selected for kanamycin resistance and further confirmed by Northern blot analysis. The *al3* mutant (Salk_139843c) contains a T-DNA insertion in the fifth exon of the *AtAL3* gene, the *al7-1* mutant (Salk_127650), and *al7-2* mutant (Salk_127657) contains a T-DNA insertion in the first exon of the *AtAL7* gene. T-DNA insertions were confirmed by PCR using the primers (5′-TGACCCTGGTTAGGGTTTCTC-3′ and 5′-TGGAACCACTTCTCACAAAGG-3′ for *al3*, 5′-TGAGGATAAAAGCATCAACGC-3′ and 5′-AGAAAGCCAAAATCTTTTGGG-3′ for *al7-1*, and 5′- TGAGGATAAAAGCATCAACGC-3′ and 5′-TGAGACCAGCTCTACGACCAC-3′ for *al7-2*.).

### Northern Blot and RT-qPCR

Total RNA was isolated by phenol/chloroform extraction and LiCl precipitation. For the Northern blot analysis, 20 µg of total RNA was mixed with two times the volume of RNA denaturing sample buffer (Embitec) and was incubated in a 68°C water bath for 15 minutes and ice bath for 5 minutes. Then, the mixture was separated on formaldehyde (18.6% v/v) agarose gels (1.5% m/v), stained with ethidium bromide, and blotted to nylon membrane. The nylon membranes were hybridized with [α-^32^P]dATP-labelled *AtAL7* specific probes in PerfectHyb plus hybridization buffer (Sigma-Aldrich) at 68°C for 16 hours. The membrane was then washed for 10 minutes twice with 2×SSC (1×SSC is 0.15 M NaCl and 0.015 M sodium citrate) and 1% SDS and for 10 minutes with 0.1×SSC and 1% SDS at 68°C. For transformant screening, 2 µg of total RNA of transgenic plants was prepared for Northern blot analysis. DNA fragments for *AtAL7* (At1g14510) probes were PCR amplified from *A. thaliana* reverse transcription products with the following primers: 5′-GCGGATCCATGGAAGGAATTCAGCATCCT-3′, 5′-GCGAGCTCTCAGGCTTTCATTTTCTTGCT-3′, and 5′-GCGGTACCGGCTTTCATTTTCTTGCTGGT-3′.

For RT-qPCR, total RNA extracted by the use of RNAiso Plus kit (Takara). According to the method described by Michael [42], the first-strand cDNA was synthesized from 1.5 µg DNase-treated RNA in a 20 µL action volume using M-MuLV reverse transcriptase (Fermentas, now Thermo Scientific, http://www.thermoscientificbio.com) with oligo(dT)18 primer. Relative transcripts levels were determined using the iCycler IQ Real-time PCR Detection System (Bio-Rad, USA) according to the manual QuantiTect SYBR Green PCR kit and analyzed by icycler real-time detection system software (version 3.0). *ACTIN2* was used as a control. Gene-specific primers used to detect transcripts are listed in Table S1 in File S1.

## Results

### Co-evolution of Separate Domains Among Four Groups of AL Proteins

**Figure 1:**
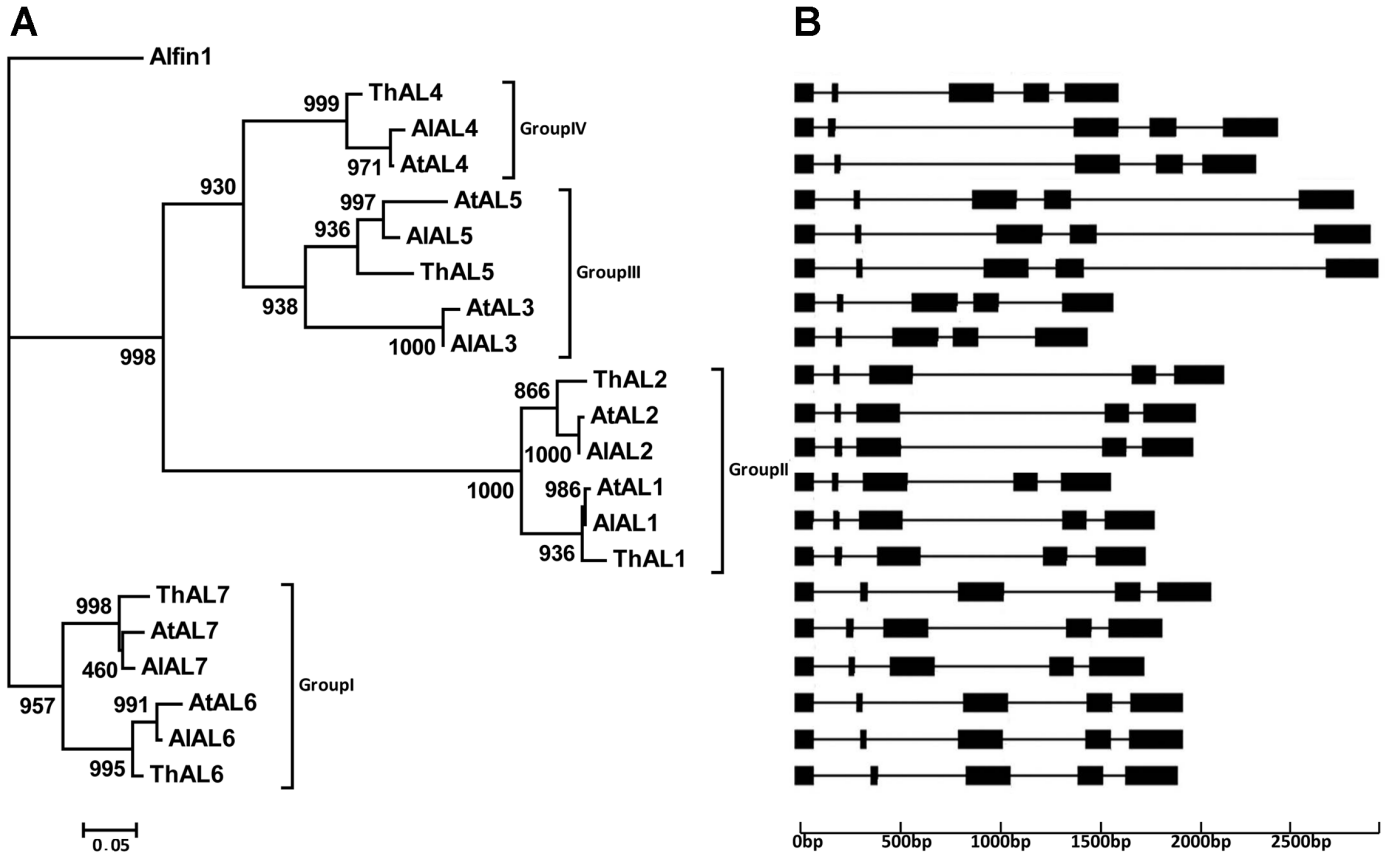
Phylogenetic analysis and gene structure of AL proteins in *A.* *lyrata*, *A. thaliana*, and *T. halophila*. A. The tree was constructed from a complete alignment of 21 AL proteins by maximum likelihood (ML) method with bootstrapping analysis (1000 reiterations). In ML methods, the evolutionary model was JTT+G which determined by ModelGenerator v0.84 software. B. The horizontal lines indicated the position of introns and the black boxes represented the location of exons.

The phylogenetic reconstruction of the twenty AL proteins observed in the three species clearly separated into four distinct groups: I, II, III and IV (Fig. 1A), using ML estimation and Alfin1 of alfalfa as an out-group with an in-group consisting of seven, seven, and six AL protein sequences of *A. thaliana*, *A. lyrata*, and *T. halophila*, respectively. All genes of the members on the tree had five exons separated by four introns (Fig. 1B). All the clades except AL3 clade had three orthologs from *A. thaliana, A. lyrata*, and *T. halophila*, indicating that the orthologs of each clade may have originated from one gene in an ancestral species.

**Figure 2:**
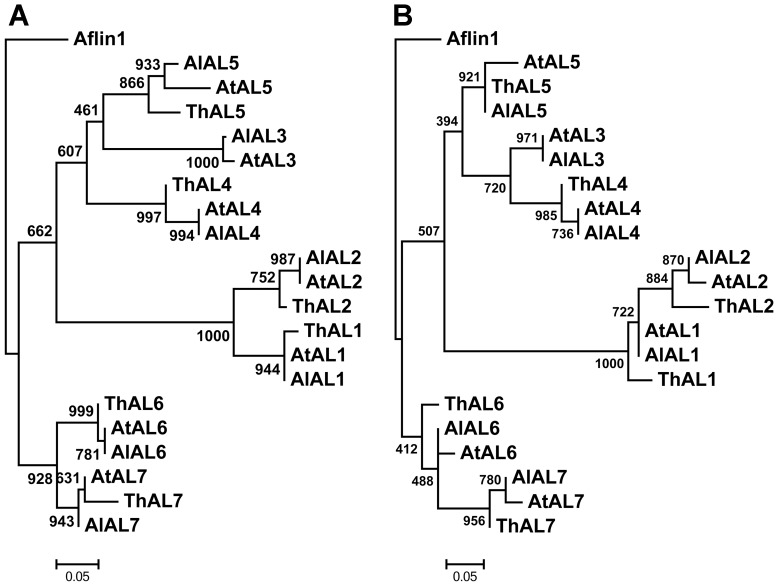
Phylogenetic trees of the DUF3594 domain (A) and PHD finger (B) sequences. These trees were inferred by the neighbor-joining method after alignment of the DUF3594 and PHD finger domain amino acid sequences of the 20 proteins that contained both DUF3594 domain and PHD finger in *A. lyrata*, *A. thaliana*, and *T. halophila*.

Additionally, all of the AL proteins possessed both the DUF3594 and PHD-finger domains (Figure S1 in File S1), and separate phylogenetic reconstruction of the two domain recovered similar evolutionary histories as the entire AL sequence (Fig. 2). The phylogenetic reconstruction for the PHD finger sequences were less resolved (Fig. 2B) and the only direct conflict between the two trees is not strongly supported. This evolutionary congruence indicates that little or no recombination has occurred within and among the four groups of *AL* genes. Further, the pairwise distances between sequences of the DUF3594 domain were strongly correlated with the pairwise distances among PHD-finger domain sequences and their inter-domain, using either direct linear correlation or a Mantel test (r values reported respectively; r = 0.745 and 0.7933 for the DUF3594 domain and inter-domain; r = 0.7850 and 0.7038 for the PHD-finger domain and inter-domain, and r = 0.8522 and 0.8043 for the DUF3594 and PHD-finger domains; p<0.001 for all correlations). Notably, the correlation coefficients of both tests were higher between DUF3594 and PHD-finger sequences than for comparisons with the inter-domain sequence. The two domains in these genes are clearly evolving together and have similar histories.

### Evolutionary Patterns of *AL* Genes in *A. thaliana*

Eight pairs of paralogous genes (three for *A. thaliana*, three for *A. lyrata*, and two for *T. halophila*) were identified at the tips of the phylogenetic tree (Fig. 1A). We tested the segmental duplication events and compared the flanking 10 protein-coding genes of both the *AL* gene of *A. thaliana* and its paralogs. There were three pairs of genes flanking *AtAL1* on chromosome 5 and *AtAL2* on chromosome 3 which showed high conservation (Table 1, Table S2 in File S1), suggesting that these two *AL* genes were formed through segmental duplication in *A. thaliana*. For other pairs of *AL* paralogous genes in *A. thaliana,* there was no evidence supporting their origin from duplicated blocks. These results revealed that several members of the *AtAL* family arose through segmental duplications. Also, we searched the orthologs of the three pairs of flanking genes existing beside *AtAL1* and *AtAL2* in *A. lyrata* and *T. halophila* (Table 1), and the highly conserved hits were detected, indicating that similar segmental duplication event could have occurred in the ancestor of the three species.

**Table 1:** The matched paralogs of the genes in the flanking region of duplicated *AL* genes in three species.

Taxon	Gene 1	Gene 2	Identity	Similar	E-value	GC3 of G1	GC3 of G2
*Arabidopsis lyrata*	AlAL1	AlAL2	87.7%	93.9%	6.1E-85	46.28%	42.34%
	487361	928831	77.7%	89.9%	2.1E-125	48.79%	47.45%
	487353	478456	79.3%	89.6%	1.2E-120	41.97%	40.27%
	487349	928823	73.7%	87.3%	4.4E-58	52.02%	50.20%
		Average	79.6%	90.2%		47.22%	45.06%
***Arabidopsis thaliana***	**AtAL1**	**AtAL2**	**87.2%**	**94.2%**	**3.0E-89**	**45.45%**	**44.13%**
	**AT5G05600**	**AT3G11180**	**74.7%**	**88.9%**	**2.8E-112**	**48.66%**	**51.49%**
	**AT5G05580**	**AT3G11170**	**80.5%**	**89.2%**	**6.0E-120**	**45.43%**	**39.37%**
	**AT5G05550**	**AT3G11100**	**72.4%**	**88.6%**	**3.0E-55**	**53.44%**	**50.00%**
		**Average**	**78.7%**	**90.2%**		**48.25%**	**46.25%**
*Thellungiella halophila*	ThAL1	ThAL2	87.2%	94.2%	3.0E-89	51.54%	45.71%
	Thhalv10013861m	Thhalv10022012m	75.1%	90.1%	2.6E-112	50.81%	51.58%
	Thhalv10013625m	Thhalv10020755m	81.6%	90.7%	7.8E-118	45.69%	46.47%
	no hit	no hit					
		Average	81.3%	91.7%		49.35%	47.92%

We used GeneConv software to investigate the possible gene conversion events that might have occurred during the evolution of *AL* genes, but no such event was detected. Peculiarly, the phylogenetic tree Group I contained 8 members, but only AtAL3 and AlAL3 have no ortholog in *T. halophila* (Fig. 1). We detected 20 genes located in the flanking region of *AtAL3* on chromosome 3 in the NCBI database and found 16 pseudogenes (Table S3 in File S1). It has also been reported previously that AtAL3 lacked the key conserved Tyr residue on its PHD-finger domain [16]. All of these evidences suggest that a gene loss event had occurred in the *AL* gene family during the divergence between *A. thaliana* and *T. halophila* or after the divergence of *T. halophila* from *Arabidopsis* lineage.

### Testing for Selection in the *AL* Genes of *A. lyrata*, *A. thaliana*, and *T. halophila*

In order to detect the evolutionary driving forces in the divergence of the *AL* gene family, we used the BEB method to predicted positively selected codon sites on *AL* genes of *T. halophila*, *A. thaliana*, and *A. lyrata*. Both site-specific models and branch-site models were compared to evaluate the evolutionary forces for the former assuming variable selective pressures among sites and the latter examining the selective pattern among branches in the phylogeny. As shown in Table 2, the site-specific models failed to detect any site under positive selection, but showed signs of selective sweep on most of the *AL* gene sites (89.23% sites with ω<1). Moreover, we implemented the branch-site model to detect whether the positive selection acted on some sites of specific clades in *AL* gene phylogeny. As summarized in Table 3, four amino acids were identified by BEB analysis as candidates for positively selected sites with Bayesian posterior probability >0.95. With one exception in the PHD-finger domain of AL7 (alignment position 233K in Figure S1 in File S1), the amino acids were located in the DUF3594 domain of AL1, AL2, and AL6 protein (alignment positions 33T, 37K and 35V, respectively, in Figure S1 in File S1).

**Table 2:** Tests for selection among codons of AL proteins using site models.

Site-specific models	lnL^a^	Parameter estimations	?^2^ b	Positively Selected Sites
M0 (one-ratio)	−5734.773	ω = 0.091		Not allowed
M1a (nearly neutral)	−5673.941	p_0_ = 0.8923,p_1_ = 0.1077ω_0_ = 0.0723, ω_1_ = 1.0000		Not allowed
M2a (positive selection)	−5673.941	p_0_ = 0.8923,p_1_ = 0.1077,p_2_ = 0.0000ω_0_ = 0.0723, ω_1_ = 1.0000, ω_2_ = 33.741	0	None
M3 (discrete)	−5607.533	p_0_ = 0.5051,p_1_ = 0.3767,p_2_ = 0.1182ω_0_ = 0.0088, ω_1_ = 0.1350, ω_2_ = 0.4545	254.48(P<0.01)	None
M7 (beta)	−5607.627	p = 0.3872,q = 3.0408		Not allowed
M8 (beta & ω)	−5607.630	p_0_ = 0.99999,P = 0.3871,q = 3.0403(p_1_ = 0.00001), ω = 3.4633	0	None

a The proportion of sites (p0, p1, etc.) estimated to have ω_0_, ω_1_, etc.

b 2(l_1_– l_2_).

**Table 3:** Parameter estimation and likelihood ratio tests for the branch-site models.

Branch-site models	lnL^a^	Parameter estimations	?^2^ b	Positively Selected Sites
**I**	***AL6*** −5444.364	P_0_ = 0.865 P_1_ = 0.095 P_2a_ = 0.036 P_2b_ = 0.004ω_b0_ = 0.068 ω_b1_ = 1.000 ω_b2a_ = 0.068 ω_b2b_ = 1.000ω_f0_ = 0.068 ω_f1_ = 1.000 ω_f2a_ = **999.000**ω_f2b_ = **999.00**	5.909	35V(0.964)
	***AL7*** −5445.564	P_0_ = 0.871 P_1_ = 0.112 P_2a_ = 0.015 P_2b_ = 0.002ω_b0_ = 0.069 ω_b1_ = 1.000 ω_b2a_ = 0.069 ω_b2b_ = 1.000ω_f0_ = 0.069 ω_f1_ = 1.000 ω_f2a_ = **998.997**ω_f2b_ = **998.997**	3.100	233K(0.994)
**II**	***AL1*** −5443.461	P_0_ = 0.858 P_1_ = 0.095 P_2a_ = 0.036 P_2b_ = 0.004ω_b0_ = 0.068 ω_b1_ = 1.000 ω_b2a_ = 0.068 ω_b2b_ = 1.000ω_f0_ = 0.068 ω_f1_ = 1.000 ω_f2a_ = **13.646**ω_f2b_ = **13. 646**	4.698	33T(0.978)
	***AL2*** −5443.861	P_0_ = 0.845 P_1_ = 0.103 P_2a_ = 0.046 P_2b_ = 0.006ω_b0_ = 0.067 ω_b1_ = 1.000 ω_b2a_ = 0.067 ω_b2b_ = 1.000ω_f0_ = 0.067 ω_f1_ = 1.000 ω_f2a_ = **33.525**ω_f2b_ = **33.525**	3.994	37K(0.988)
**III**	***AL3*** −5448.462	P_0_ = 0.809 P_1_ = 0.106 P_2a_ = 0.076 P_2b_ = 0.010ω_b0_ = 0.068 ω_b1_ = 1.000 ω_b2a_ = 0.068 ω_b2b_ = 1.000ω_f0_ = 0.068 ω_f1_ = 1.000 ω_f2a_ = 1.000 ω_f2b_ = 1.000	0	Not found
	***AL5*** −5449.079	P_0_ = 0.888 P_1_ = 0.112 P_2a_ = 0.000 P_2b_ = 0.000ω_b0_ = 0.070 ω_b1_ = 1.000 ω_b2a_ = 0.070 ω_b2b_ = 1.000ω_f0_ = 0.070 ω_f1_ = 1.000 ω_f2a_ = 1.000 ω_f2b_ = 1.000	0	Not found
**IV**	***AL4*** −5449.079	P_0_ = 0.888 P_1_ = 0.112 P_2a_ = 0.000 P_2b_ = 0.000ω_b0_ = 0.070 ω_b1_ = 1.000 ω_b2a_ = 0.070ω_b2b_ = 1.000ω_f0_ = 0.070 ω_f1_ = 1.000 ω_2a_ = 1.000 ω_f2b_ = 1.000	0	Not found

a Likelihood of the model.

b 2(l_1_–l_0_).

### Comparisons of Nucleotide Variation and Synonymous Codon Usage in the *AL* Gene Family of Three Species

**Figure 3:**
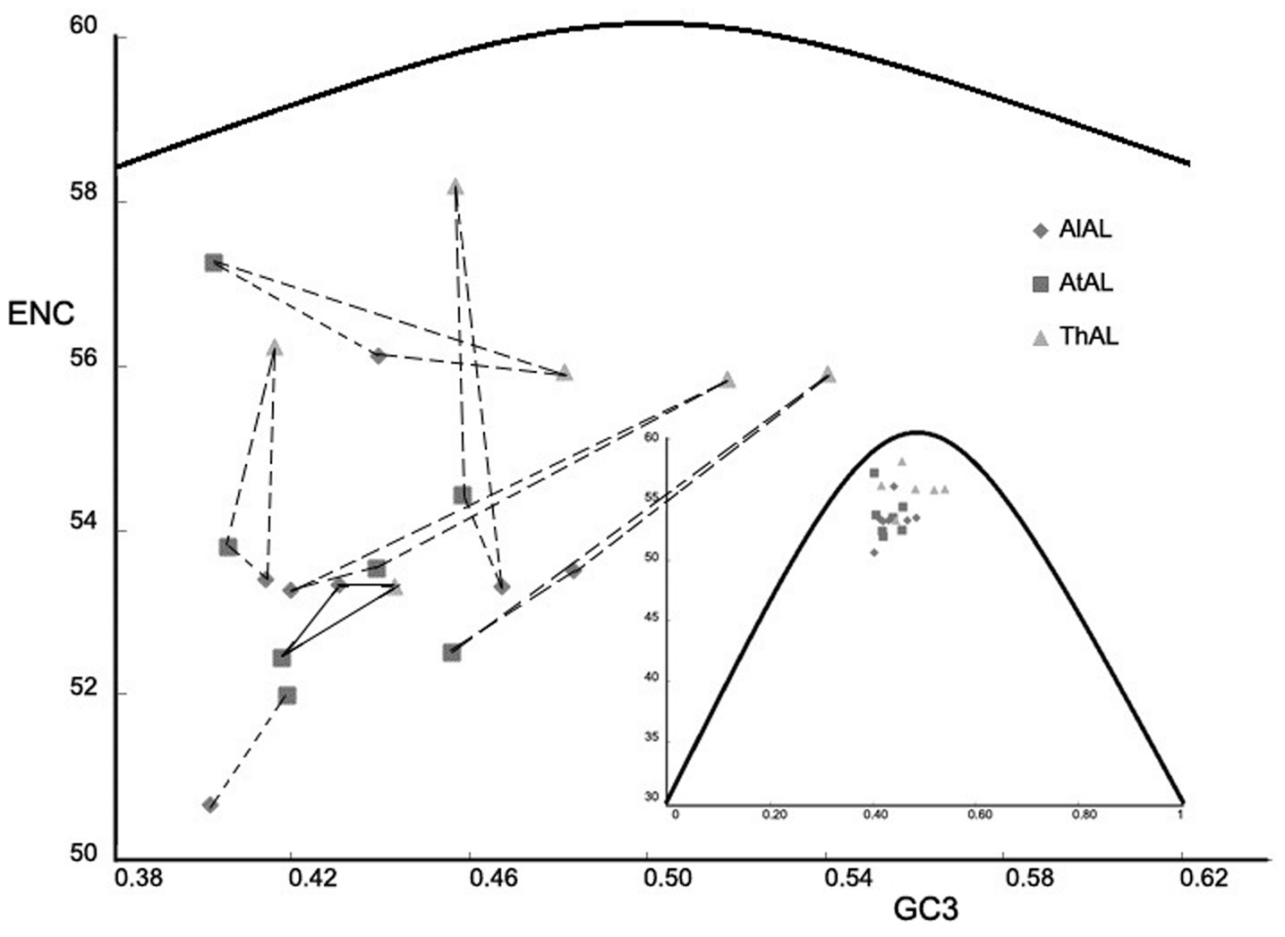
Effective number of codons (ENC) used in a gene versus the percent GC at third codon positions (GC3) for *AL* genes of *A.* *lyrata*, *A. thaliana*, and *T. halophila*. The solid curve was the expectation of the ENC under the assumption of no selection on codon usage. The three markers connected with solid line indicate the values of *AL7* genes in *A. lyrata*, *A. thaliana*, and *T. halophila*, and the other three markers connected with dotted line indicate the values of other *AL* genes in *A. lyrata*, *A. thaliana*, and *T. halophila*.

The observed differences in GC content at the three positions and the effective number of codons (ENC) among the AL members of *A. lyrata*, *A. thaliana*, and *T. halophila* indicated a narrow range of GC3 and codon usage patterns. Mean GC3 content and ENC were significantly different between the members of *A. thaliana* and *T. halophila*, with values of 0.428 and 53.7 for *AtAL*s and 0.476 and 55.9 for *ThAL*s, respectively (Table S4 in File S1). Further, we detected that the GC3 difference primarily came from the DUF3594 domain rather than the PHD finger and inter-domain, and a significant difference existed between the domains of *A. lyrata* and *T. halophila*. In addition, the possible duplication fragments of *A. lyrata* and *T. halophila* also presented significant GC3 content differences (Table 4). GC3 and ENC values charted for all *AL* genes were significantly different from the expected ENC curve which represents the null hypothesis that GC3 bias was entirely due to mutation rather than selection, indicating that selection was likely driving biased codon usage. Overall, the *AL* genes are tightly clustered in a narrow range of GC3 content and ENC value. The only visible trend was that the points for *ThAL*s were more loosely clustered than any other *AL* genes of the two species and lay nearest to the expected value (Fig. 3).

**Table 4:** Summary of the gene length, GC content, and codon usages P-value among three species’ *AL* gene.

Taxon	AL gene length			AL Gene cDNA						DUF3594 inter-domain PHD finger					
	Gene	Extron	Intron	GC	GC1	GC2	GC3	ENC	CAI	GC3	ENC	GC3	ENC	GC3	ENC
Al vs At	0.43	1.00	0.41	0.78	1.00	0.85	0.65	0.71	0.60	0.78	0.39	0.74	0.53	0.62	0.78
Al vs Th	0.62	0.52	0.59	0.07	0.78	0.53	**0.05**	**0.01**	0.15	**0.04**	0.69	0.36	0.48	0.31	0.87
At vs Th	0.22	0.52	0.20	0.11	0.78	0.62	**0.02**	**0.03**	0.06	**0.02**	0.23	0.49	0.20	0.16	0.70

### Overexpression of *AtAL7* Suppressed Plant Root Growth Under Normal and Saline Conditions

**Figure 4:**
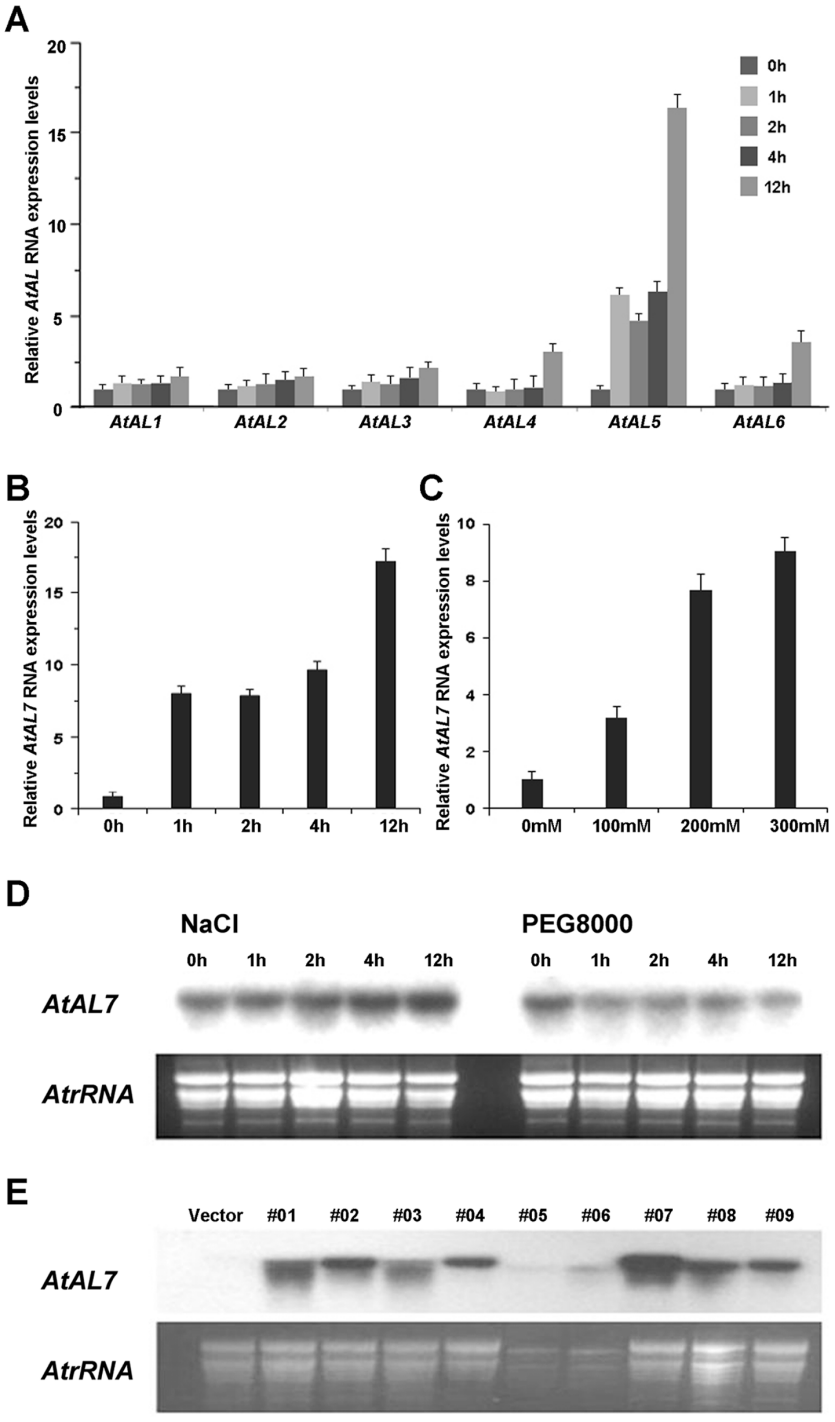
Expression patterns of *AL* genes in *A.* *thaliana* and expression of *AL7* in independent transgenic lines. A. RT-qPCR analyses of *AtAL1∼6* genes responding to salt stress. B. RT-qPCR analyses of *AtAL7* gene responding to salt stress. RNA samples were prepared from 3-week-old wild-type plants at the given times after treatment with salt (300 mM NaCl). C. RT-qPCR analyses of *AtAL7* genes responding to different salt concentrations in *A. thaliana*. RNA samples were prepared from 3-week-old wild-type plants after treatment with 100 mM NaCl, 200 mM NaCl, and 300 mM NaCl solutions for 4 hours. Error bars show standard deviations from three independent RNA extractions. D. Northern blot analysis of *AtAL7* genes responding to abiotic stresses. Each lane was loaded with 20 µg total RNA isolated from 21-day-old seedlings of *A. thaliana*. E. Northern blot analyses of *AtAL7* expression in *AtAL7* overexpression plants. RNA samples were prepared from leaves of nine 21-day-old lines of *AtAL7* overexpression plants. A 2 µg portion of RNA was separated on an agarose-formaldehyde gel.

Previous study reported that *Alfin1* functions in salt tolerance of alfalfa [19]. Here, we examined the induced expression of *AL* genes in *A. thaliana* under treatments with NaCl solution by quantitative real-time PCR (RT-qPCR). As shown in Figure 3, *AtAL5* (Fig. 4A) and *AtAL7* (Fig. 4B) expression were strongly up-regulated by 300 mmol/L NaCl solution treatment, and the levels of *AtAL7* transcripts increased with prolonged treatment time (Fig. 4B) as well as increased NaCl concentration (Fig. 4C). Northern blot results also indicated that *AtAL7* was induced by salt stress and inhibited by osmotic stress (Fig. 4D). It was also shown that only *AL7* from the seven *AL* gene clades was under positive selection on the PHD-finger domain (Figure S1 in File S1), and the three members on this branch showed the smallest difference in codon bias, as shown in the graph among the seven clusters (Fig. 3). We therefore selected the *AtAL7* gene for further functional analysis.

**Figure 5:**
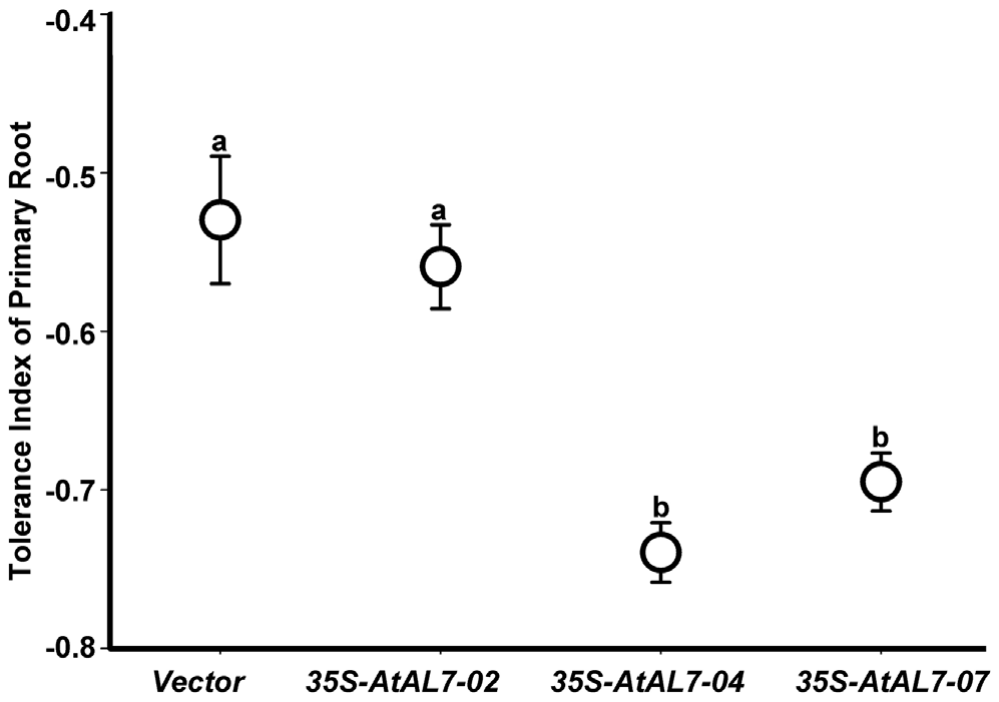
Response of *35S-AtAL7 Arabidopsis* lines to NaCl. The tolerance index of primary root of different abiotic stress treated vector and *35S-AtAL7* plants was compared with control plant (0 mM NaCl).Values graphed are means ±SE (n = 12). a and b, one way ANOVA with Bonferroni multiple comparison test significant at P≤0.01 between two of vector, *35S-AtAL7-02*, *35S-AtAL7-04*, and *35S-AtAL7-07* plants.

To determine the physiological role of *AtAL7* in transgenic *A. thaliana* under salt stress, we generated transgenic *A. thaliana* plants overexpressing the *AtAL7* gene under the CaMV 35S promoter. Among the nine randomly selected primary T1 transformants from the pool of forty two transformants (Fig. 4E), we selected three individual overexpressing lines of *AtAL7* using Northern blot analysis for collecting the T3 generation seeds. Then we further compared the root morphology of the vector, *35S-AL7-2*, *35S-AL7-4*, and *35S-AL7-7* plants grown on MS medium with 0 mM, 150 mM, and 200 mM NaCl (Figure S2 in File S1). Three *35S-AL7* transgenic lines exhibited shorter root lengths than vector plants growing on all culture media (Fig. 5). Relative primary root lengths of the *35S- AL7* transgenic lines were significantly shorter than the vector plant seedlings on MS agar plates with 200 mM NaCl, that suggesting over-expression of *AtAL7* in *A. thaliana* could reduce plant tolerance to salt stress.

### The T-DNA Insertion Mutants of *AtAL7* Enhanced Plant Root Growth Under Normal and Saline Conditions

**Figure 6:**
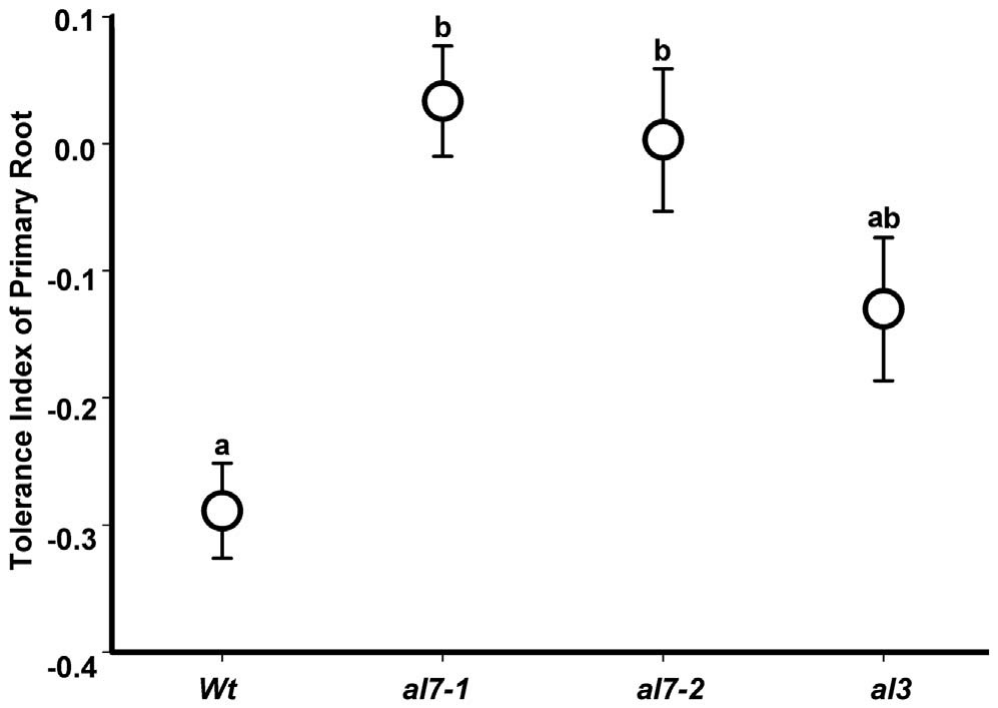
Response of *Arabidopsis AL* mutants to NaCl. The tolerance index of primary root of different abiotic stress treated wild type, *al7-1*, *al7-2*, and *al3* plants was compared with control plant (0 mM NaCl). Values graphed are means ±SE (n = 12). a and b, one way ANOVA with Bonferroni multiple comparison test significant at P<0.01 between two of wild type, *al7-1*, *al7-2*, and *al3* plants.

To confirm the negative role of *AtAL7* in plant salt tolerance, we identified two T-DNA mutants (Figure S3 in File S1). Both the *al7-1* mutant (Salk_127650) and *al7-2* mutant (Salk_127657) contain a T-DNA insertion in the first exon at the 5′ untranslated region of the *AtAL7* gene. Additionally, we identified another T-DNA mutant of *AtAL3* gene as a control. The *al3* mutant (Salk_139843c) contains a T-DNA insertion in the fourth intron of the *AtAL3* gene. Homozygous mutant plants were identified by PCR with *AtAL7* or *AtAL3* specific primers. We further compared the root morphology of the *al3* mutant, *al7-1* mutant, *al7-2* mutant, and wild type plants grown on MS medium with 0 mM and 150 mM NaCl (Figure S4 in File S1). The T-DNA insertion mutants of *AtAL7* exhibited longer root lengths than *al3* mutant and wild type plants growing on all culture media (Fig. 6), suggesting that *AtAL7* could play a negative role in *A. thaliana* resistance to the salt stress.

## Discussion

It had long been known that AL proteins play important roles in regulating signals of plant salt tolerance [14], [19], [43]. The AL transcription factor is a two-domain protein with the ability to bind to the highly methylated forms of histones and functions in plant salt tolerance [16], [44]. Previous work has shown that the co-evolution might have taken place among the protein family members which have more than two domains [45]–[47]. Here the two phylogenetic trees, one for each domain (Fig. 2 A and B), exhibited all three distinct groups and each group contained the same members in the PHD-finger and DUF3594 domain trees, implying a co-evolutionary relationship between the two domains in AL trans-acting factors, similar to that with the tubby like proteins [48]. Correlations between both domains also supported their co-evolution according to the method by Goh *et al*. [30] and Mantel test. Moreover, results of branch site model analysis detected more positive selection sites on the DUF3594 domain than the PHD-finger and linking region (Table 3), and the significant difference in codon usage bias between *T. halophila* and *A. lyrata* or *A. thaliana* occurred on the DUF3594 domain rather than the PHD-finger domain (Table 4). These findings suggest that functional divergences of AL proteins primarily came from the DUF3594 domain.

DUF3594 domain, approximately 140 amino acids in length, is functionally uncharacterized in eukaryotes, while PHD-finger domain is thought to facilitate protein-protein interaction with tri- and dimethylation of histone H3 at lysine 4 (H3K4me3/2) [13], [16], [49]. In plants, Alfin1 containing a canonical PHD finger was reported to bind to promoter elements of *MsPRP2* gene [14]. Another study suggested that Alfin1 is a transcription co-activator but not a transcription activator [16]. Here, we detected three positively selected sites on DUF3594 domain and one site on PHD-finger domain, which implied that possible adaptive evolution occurred on these two domains. It is known that *AtAL3* lacks the key conserved Tyr residue on its PHD-finger domain and does not bind to H3K4me3 [16]. In our gene function analysis, however, the mutant plants of *AtAL3* displayed slightly enhanced salt tolerance as the mutants of *AtAL7* (Fig. 6), suggesting that the possible adaptive evolution tend to occurred on DUF3594 domain rather than PHD-finger.

From the evolutionary viewpoint, gene duplication, gene transfer and gene losses play key roles in the evolution of gene families and accelerate the turnover of gene birth and death of the family members’ evolution [50], [51]. There are three types of gene duplication: transposition events, segmental duplication, and tandem duplication [52]. In our analysis, we found that *AtAL1* and *AtAL2*, with three pairs of high conserved genes in its flanking region (Table 3), had expanded through segmental duplication in the three species. In contrast, gene loss has been put forward as a common response to changes from duplicated genome segments in *A. thaliana* [53], and was also observed in the triplicated genome segments of *Brassica oleracea* [54]. Here, we identified that only *AtAL3* and *AlAL3* have no orthologous genes in *T. halophila* (Fig. 1), multiple genes beside *AtAL3* were pseudogenes, and a previous study reported that *AtAL3* lacked the key conserved Tyr residue on its PHD-finger domain [16], implying the *AL* gene loss event could have occurred in the ancestral genome of the *Thellungiella* lineage.

Multiple studies on the duplication processes in the molecular evolution of plant regulatory genes have argued that trans-acting factors often have increased the rates of non-synonymous substitutions compared with structural genes [6], [7], [55], [56]. In several cases it is clear that neutrally evolving regions play important roles in protein function [3], [57]–[59]. In this study we used site-specific models and branch-site models to detect positive selection among the *AL* genes of three species. The latter models predicted one site as positively selected for *AL1*, *AL2*, *AL6*, and *AL7* of seven branches respectively (Table 4), suggesting that positive selection has operated on *AL* genes in the three species during their evolution. Moreover, we also analyzed the codon usage bias which reflects a balance between mutational biases and natural selection for translational optimization [60]–[62]. ENC values and Mean GC3 statistics indicate significant differences between *AL* genes of *A. thaliana* and *T. halophila* (Table 3), and differences also may exist between the *AL* genes of *A. lyrata* and *T. halophila* (Table 3). In Figure 3, the gene cluster of *T. halophila* was obviously separated from the clusters of *A. thaliana* and *A. lyrata*, suggesting the differentiation of biased codon usage between *T. halophila* and the other two species had already occurred.

## Supporting Information

File S1 Figure S1 in File S1. Amino acid sequence alignment for 20 AL proteins by ML methods with bootstrapping analysis (1000 reiterations). The DUF3594 domain and PHD-finger are indicated by yellow and blue boxed letters. The positively selected codon sites are indicated by red arrows. The amino acids in red box display the altered key site as Lee *et al* [16]. Figure S2 in File S1. Phenotype response of *35S-AtAL7 A. thaliana* lines to normal condition (left), 150 mM NaCl (middle), and 200 mM NaCl (right). Seedlings of vector (upper left), *35S-AtAL7-02* (upper right), *35S-AtAL7-04* (bottom left), and *35S-AtAL7-07* (bottom right) transgenic lines were germinated on a 1/2MS agar plate for 3 days, then transferred to another MS agar plate supplemented with 150 mM NaCl (middle) and 200 mM NaCl (right) for 5 days. Figure S3 in File S1. Diagram of *AtAL3* and *AtAL7* and their T-DNA insertion mutants. Figure S4 in File S1. Phenotype response of *A. thaliana AL* mutants to normal condition (left) and 150 mM NaCl (right). Seedlings of wild type (upper left), *al7-1* (upper right), *al7-2* (bottom left), and *al3* (bottom right) mutants were germinated on a 1/2MS agar plate for 3 days, then transferred to another 1/2MS agar plate supplemented with 150 mM NaCl (right) and without (left) for 4 days.
(DOC)
