# Supplementary material for: Molecular Evolutionary Analysis of the Alfin-Like Protein Family in Arabidopsis lyrata, Arabidopsis thaliana, and Thellungiella halophila
Source: PLoS One. 2013 Jul 1;8(7):e66838. doi: 10.1371/journal.pone.0066838 (PMC3698079; doi:10.1371/journal.pone.0066838)

Supplemental Table S1. Primers used for RT-qPCR.

| Gene | Primers (5’->3’) |
| --- | --- |
| *AtAL1* | AGCACGCCGAAACTAATGGA  TTAGTGCAGCAAGAAGGGCA |
| *AtAL2* | GGAGCCCGTCTTAATCGCAA  TTGAGCTCTTTGTCTGCCCG |
| *AtAL3* | AAGGTCGTCGTACTGCCATT  TCAGAGAGCCCATCCCTAGC |
| *AtAL4* | CCGTGCCAAGTTCTCAAAGC  GCTGCATAGCTCTCACCACA |
| *AtAL5* | TGGCTTTCTCTCGTTGCTGT  GCGCTTCCTGTCAGCTCTAT |
| *AtAL6* | AGCAAACCTAGTGGCGTCAA  TGTCTCCACAAGCACCACAA |
| *AtAL7* | GCGACTTTCGTGGTCGTAGA  TGATGCCTAACGCTGGTTCA |
| *ACTIN2* | TGTGCCAATCTACGAGGGTTT  TTTCCCGCTCTGCTGTTGT |

Supplemental Table S2. Summary of the gene locus name, gene location, gene annotation, and segmental length of genes in the flanking region of duplicated *AL* genes in *A. lyrata*, *A. thaliana*, and *T. halophila*.

| **Taxon** | **Locus name** | **Gene location** | **Gene annotation** | **Segmental length** |
| --- | --- | --- | --- | --- |
| *A. lyrata* | 487363 | scaffold_6: 2066127 - 2068442 | AlAL1 | 2068442 ~ 2044415  24027bp |
|  | 487361 | scaffold_6: 2059268 - 2061956 | 2OG-Fe(II) oxygenase superfamily |
|  | 487353 | scaffold_6: 2044415 - 2046778 | omega-3 fatty acid desaturase |
|  | 487349 | scaffold_6: 2016127 - 2017487 | Transcription factor GT-2 and related proteins | 1360bp |
|  | 478459 | scaffold_3: 4492884 - 4495404 | AlAL2 | 4495404 ~ 4483195  12209bp |
|  | 928831 | scaffold_3: 4487523 - 4491344 | 2OG-Fe(II) oxygenase superfamily |
|  | 478456 | scaffold_3: 4483195 - 4485719 | omega-3 fatty acid desaturase |
|  | 928823 | scaffold_3: 4457256 - 4458175 | Transcription factor GT-2 and related proteins | 919bp |
| *A. thaliana* | AT5G05610 | Chromosome 5, NC_003076.8 (1676932..1679104) | AtAL1 | 1679104 ~ 1664085  15019bp |
|  | AT5G05600 | Chromosome 5, NC_003076.8 (1672120..1674739) | leucoanthocyanidin dioxygenase like protein  2OG-Fe(II) oxygenase family protein |
|  | AT5G05580 | Chromosome 5, NC_003076.8 (1664085..1666531) | FAD8, omega-3 fatty acid desaturase |
|  | AT5G05550 | Chromosome 5, NC_003076.8 (1639032..1640606) | sequence-specific DNA binding transcription factor | 1574bp |
|  | AT3G11200 | Chromosome 3, NC_003074.8 (3508042..3510627) | AtAL2 | 3510627 ~ 3499801  10826bp |
|  | AT3G11180 | Chromosome 3, NC_003074.8 (3504204..3507113) | leucoanthocyanidin dioxygenase-like protein  2OG-Fe(II) oxygenase superfamily |
|  | AT3G11170 | Chromosome 3, NC_003074.8 (3499801..3502452) | FAD7, omega-3 fatty acid desaturase |
|  | AT3G11100 | Chromosome 3, NC_003074.8 (3476187..3477405) | sequence-specific DNA binding transcription factor | 1218bp |
| *T. halophila* | Thhalv10014538m | scaffold_2: 1629882 - 1632108 | ThAL1 | 1632108 ~ 1617400  14708bp |
|  | Thhalv10013861m | scaffold_2: 1625934 - 1628819 | 2OG-Fe(II) oxygenase superfamily |
|  | Thhalv10013625m | scaffold_2: 1617400 - 1619938 | omega-3 fatty acid desaturase |
|  | No hit |  |  |  |
|  | Thhalv10021406m | scaffold_13: 5835294 - 5837998 | ThAL2 | 5846134 ~ 5835294  10840bp |
|  | Thhalv10022012m | scaffold_13: 5839900 - 5842412 | 2OG-Fe(II) oxygenase superfamily |
|  | Thhalv10020755m | scaffold_13: 5843498 - 5846134 | omega-3 fatty acid desaturase |
|  | Thhalv10021383m | scaffold_13: 5866774 - 5868231 | Transcription factor GT-2 and related proteins | 1457bp |

Supplemental Table S3. Summary of the gene locus name, gene location, and gene annotation in the flanking region of *AL3* genes in *A. thaliana*.

| **Taxon** | **Locus name** | **Gene location** | **Gene annotation** |
| --- | --- | --- | --- |
|  | AT3G42820 | Chromosome 3, NC_003074.8 (14921037..14927032) | pseudo |
|  | AT3G42810 | Chromosome 3, NC_003074.8 (14919657..14920215) | pseudo |
|  | AT3G42806 | Chromosome 3, NC_003074.8 (14914008..14918667) | pseudo |
|  | AT3G42803 | Chromosome 3, NC_003074.8 (14908575..14910194) | pseudo |
|  | AT3G42800 | Chromosome 3, NC_003074.8 (14905230..14907035) | uncharacterized protein |
|  | AT3G42798 | Chromosome 3, NC_003074.8 (14901653..14902694) | pseudo |
|  | AT3G42796 | Chromosome 3, NC_003074.8 (14899844..14900608) | pseudo |
|  | AT3G42794 | Chromosome 3, NC_003074.8 (14895823..14899217) | pseudo |
|  | AT3G42792 | Chromosome 3, NC_003074.8 (14892081..14892895) | pseudo |
|  | AT3G42791 | Chromosome 3, NC_003074.8 (14888342..14891215) | pseudo |
| *A. thaliana* | **AT3G42790** | **Chromosome 3, NC_003074.8 (14877958..14879781)** | **AtAL3** |
|  | AT3G42786 | Chromosome 3, NC_003074.8 (14876476..14877255) | uncharacterized protein |
|  | AT3G42783 | Chromosome 3, NC_003074.8 (14871922..14875383, | pseudo |
|  | AT3G42780 | Chromosome: 3, NC_003074.8 (14871213..14871743) | uncharacterized protein |
|  | AT3G42770 | Chromosome 3, NC_003074.8 (14867122..14870083) | putative F-box/LRR-repeat protein |
|  | AT3G42766 | Chromosome 3, NC_003074.8 (14861485..14865389) | pseudo |
|  | AT3G42763 | Chromosome 3, NC_003074.8 (14856137..14858983 | pseudo |
|  | AT3G42760 | Chromosome 3, NC_003074.8 (14854614..14855560 | pseudo |
|  | AT3G42750 | Chromosome 3, NC_003074.8 (14853372..14854029) | pseudo |
|  | AT3G42740 | Chromosome 3, NC_003074.8 (14851876..14852568) | pseudo |
|  | AT3G42730 | Chromosome 3, NC_003074.8 (14845236..14851103) | pseudo |

Supplemental Table S4. Summary of the gene locus name, gene length, GC content, and codon usages of *AL* genes in *A. lyrata*, *A. thaliana*, and *T. halophila*. The data were collected from Phytozome Database (<http://www.phytozome.net/>) and calculated on the online platform of EMBOSS explorer (<http://emboss.bioinformatics.nl/cgi-bin/emboss>). ^a^ D_1_ (DUF3594 domain), D_2_ (inter-domain), and D_3_ (PHD finger domain).

| **Taxon** | **Gene** | **Locus name** | **Gene**  **Length** | **Extron**  **Length** | **Intron**  **Length** | **GC all** | **GC1** | **GC2** | **GC3** | **ENC** | **CAI** | **GC3**  **of D_1_^a^** | **GC3**  **of D_2_^a^** | **GC3**  **of D_3_^a^** |
| --- | --- | --- | --- | --- | --- | --- | --- | --- | --- | --- | --- | --- | --- | --- |
| *A. lyrata* | AlAL1 | 487363 | 1834 | 726 | 1108 | 46.92 | 51.75 | 42.29 | 46.70 | 53.33 | 0.73 | 44.20 | 55.00 | 46.94 |
|  | AlAL2 | 478459 | 2047 | 744 | 1303 | 45.68 | 52.59 | 42.42 | 41.99 | 53.28 | 0.72 | 39.86 | 43.18 | 46.94 |
|  | AlAL3 | 471625 | 1626 | 753 | 873 | 43.81 | 51.91 | 39.32 | 40.17 | 50.67 | 0.72 | 35.25 | 47.83 | 46.94 |
|  | AlAL4 | 941601 | 2463 | 771 | 1692 | 48.61 | 56.43 | 41.08 | 48.33 | 53.52 | 0.76 | 48.23 | 45.10 | 52.08 |
|  | AlAL5 | 484769 | 2938 | 783 | 2155 | 44.20 | 53.06 | 38.11 | 41.39 | 53.42 | 0.73 | 41.84 | 35.19 | 46.94 |
|  | AlAL6 | 935990 | 1876 | 765 | 1111 | 45.54 | 52.92 | 39.75 | 43.93 | 56.14 | 0.73 | 43.97 | 42.00 | 45.83 |
|  | **AlAL7** | **942208** | **1981** | **759** | **1222** | **46.49** | **53.78** | **42.62** | **43.04** | **53.36** | **0.73** | **42.86** | **35.42** | **51.02** |
| *A. thaliana* | AtAL1 | AT5G05610.2 | 1612 | 726 | 886 | 46.92 | 52.19 | 42.73 | 45.81 | 54.43 | 0.72 | 44.20 | 47.50 | 48.98 |
|  | AtAL2 | AT3G11200.1 | 2032 | 741 | 1291 | 46.45 | 52.81 | 42.61 | 43.91 | 53.55 | 0.72 | 42.03 | 48.84 | 44.90 |
|  | AtAL3 | AT3G42790.1 | 1491 | 753 | 738 | 44.10 | 51.49 | 38.89 | 41.88 | 52.00 | 0.72 | 40.29 | 41.30 | 46.94 |
|  | AtAL4 | AT5G26210.1 | 1656 | 768 | 888 | 47.29 | 55.42 | 40.83 | 45.61 | 52.51 | 0.74 | 42.55 | 54.00 | 45.83 |
|  | AtAL5 | AT5G20510.1 | 2856 | 783 | 2073 | 44.47 | 53.47 | 39.34 | 40.57 | 53.80 | 0.72 | 39.72 | 33.33 | 51.02 |
|  | AtAL6 | AT2G02470.1 | 1785 | 771 | 1014 | 44.48 | 53.72 | 39.42 | 40.25 | 57.27 | 0.72 | 41.43 | 33.96 | 43.75 |
|  | **AtAL7** | **AT1G14510.1** | **1984** | **759** | **1225** | **45.93** | **53.36** | **42.62** | **41.77** | **52.45** | **0.73** | **42.14** | **35.42** | **46.94** |
| *T. halophila* | ThAL1 | Thhalv10014538m | 1786 | 723 | 1063 | 47.03 | 52.38 | 43.04 | 45.65 | 58.21 | 0.73 | 48.18 | 55.00 | 59.18 |
|  | ThAL2 | Thhalv10021406m | 2195 | 735 | 1460 | 48.45 | 51.54 | 42.04 | 51.77 | 55.86 | 0.73 | 46.38 | 44.19 | 44.90 |
|  | ThAL4 | Thhalv10004808m | 2352 | 762 | 1590 | 49.51 | 54.20 | 40.34 | 54.01 | 55.91 | 0.77 | 51.06 | 54.17 | 62.50 |
|  | ThAL5 | Thhalv10014469m | 2981 | 765 | 2216 | 44.76 | 53.56 | 39.08 | 41.60 | 56.25 | 0.72 | 40.43 | 41.67 | 44.90 |
|  | ThAL6 | Thhalv10004815m | 2126 | 759 | 1367 | 46.91 | 52.10 | 40.51 | 48.10 | 55.94 | 0.74 | 52.86 | 40.82 | 41.67 |
|  | **ThAL7** | **Thhalv10008569m** | **1957** | **759** | **1198** | **47.33** | **54.20** | **43.46** | **44.30** | **53.34** | **0.75** | **42.14** | **41.67** | **53.06** |

Supplemental Figure S1. Amino acid sequence alignment for 20 AL proteins by ML methods with bootstrapping analysis (1000 reiterations). The DUF3594 domain and PHD-finger are indicated by yellow and blue boxed letters. The positively selected codon sites are indicated by red arrows. The amino acids in red box display the altered key site as Lee et al .


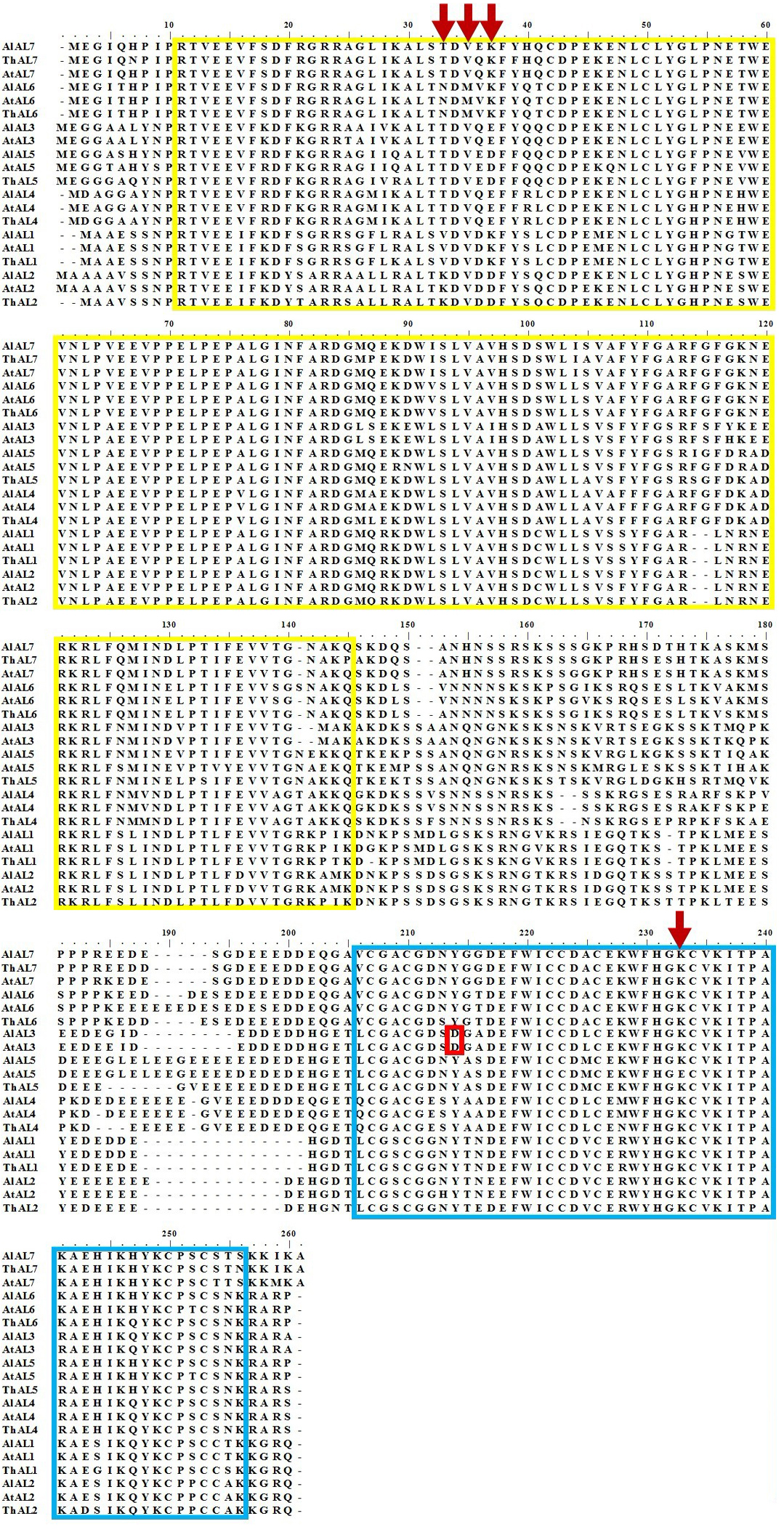


Supplemental Figure S2. Phenotype response of *35S-AtAL7* *A. thaliana* lines to normal condition (left), 150 mM NaCl (middle), and 200 mM NaCl (right). Seedlings of vector (upper left), *35S-AtAL7-02* (upper right), *35S-AtAL7-04* (bottom left), and *35S-AtAL7-07* (bottom right) transgenic lines were germinated on a 1/2MS agar plate for 3 days, then transferred to another MS agar plate supplemented with 150 mM NaCl (middle) and 200 mM NaCl (right) for 5 days.


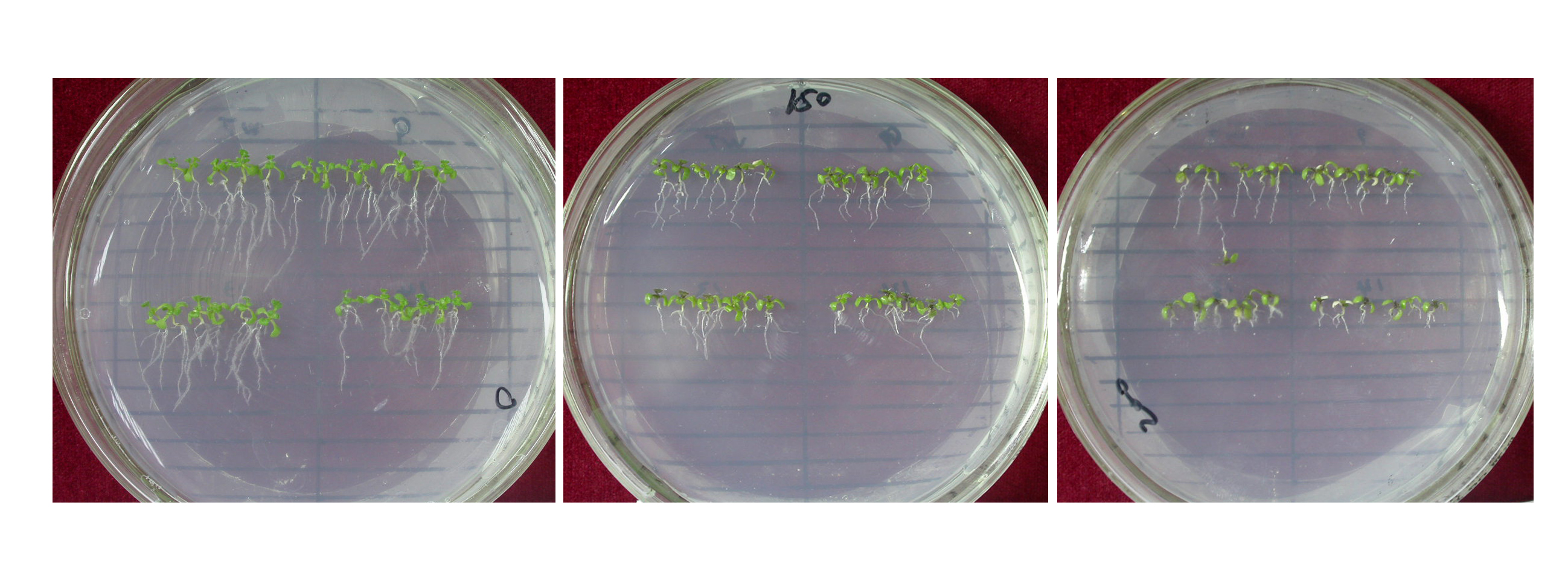


Supplemental Figure S3. Diagram of *AtAL3* and *AtAL7* and their T-DNA insertion mutants.


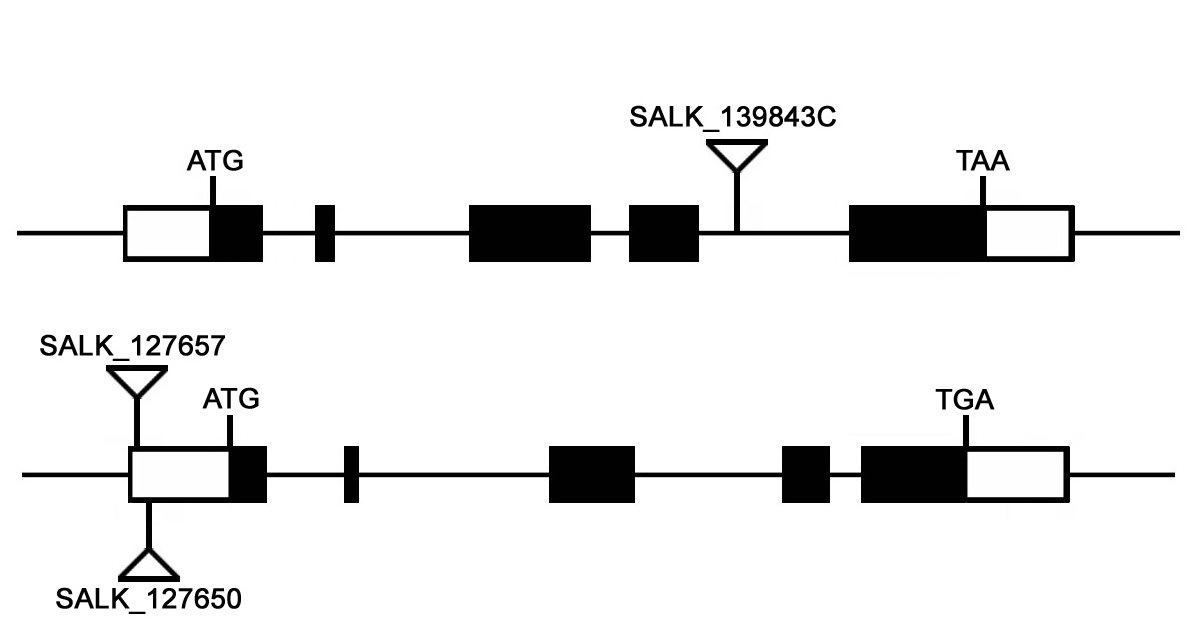


Supplemental Figure S4. Phenotype response of *A. thaliana* *AL* mutants to normal condition (left) and 150 mM NaCl (right). Seedlings of wild type (upper left), *al7-1* (upper right), *al7-2* (bottom left), and *al3* (bottom right) mutants were germinated on a 1/2MS agar plate for 3 days, then transferred to another 1/2MS agar plate supplemented with 150 mM NaCl (right) and without (left) for 5 days.


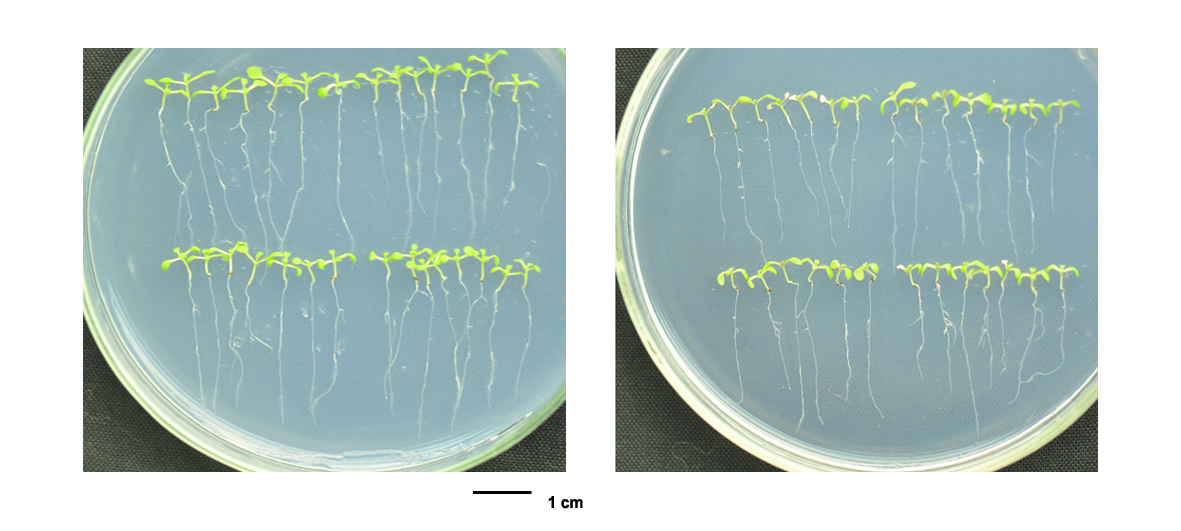

Supplement: File S1 — Figure S1 in File S1. Amino acid sequence alignment for 20 AL proteins by ML methods with bootstrapping analysis (1000 reiterations). The DUF3594 domain and PHD-finger are indicated by yellow and blue boxed letters. The positively selected codon sites are indicated by red arrows. The amino acids in red box display the altered key site as Lee et al [16]. Figure S2 in File S1. Phenotype response of 35S-AtAL7 A. thaliana lines to normal condition (left), 150 mM NaCl (middle), and 200 mM NaCl (right). Seedlings of vector (upper left), 35S-AtAL7-02 (upper right), 35S-AtAL7-04 (bottom left), and 35S-AtAL7-07 (bottom right) transgenic lines were germinated on a 1/2MS agar plate for 3 days, then transferred to another MS agar plate supplemented with 150 mM NaCl (middle) and 200 mM NaCl (right) for 5 days. Figure S3 in File S1. Diagram of AtAL3 and AtAL7 and their T-DNA insertion mutants. Figure S4 in File S1. Phenotype response of A. thaliana AL mutants to normal condition (left) and 150 mM NaCl (right). Seedlings of wild type (upper left), al7-1 (upper right), al7-2 (bottom left), and al3 (bottom right) mutants were germinated on a 1/2MS agar plate for 3 days, then transferred to another 1/2MS agar plate supplemented with 150 mM NaCl (right) and without (left) for 4 days. (DOC) [file pone.0066838.s001.doc]
